# Churg-Strauss syndrome following cessation of allergic desensitization vaccination: a case report

**DOI:** 10.1186/1752-1947-4-188

**Published:** 2010-06-22

**Authors:** Mohammad Reza Masjedi, Saeid Fallah Tafti, Ali Cheraghvandi, Nader Fayazi, Firouzeh Talischi, Bahareh Mokri

**Affiliations:** 1 Massih Daneshvari Hospital, Department of Pulmonary Medicine, Tobacco Prevention and Control Research Center (TPCRC), National Research Institute of Tuberculosis and Lung Diseases, Shahid Beheshti University of Medical Sciences, DarAbad, Tehran, Postal Code 1955841452, Iran

## Abstract

**Introduction:**

Churg-Strauss syndrome is a vasculitis of medium to small sized vessels. Diagnosis is mainly clinical with findings of asthma, eosinophilia, rhinosinusitis and signs of vasculitis in major organs.

**Case presentation:**

We present a case of a 19-year-old Persian male who developed signs and symptoms of this syndrome related to hyposensitization treatments for allergy control.

**Conclusions:**

No unifying etiology for the disease can be presented as it is found associated with environmental factors, medications, infections and is even considered a variant of asthma with predisposition to vasculitic involvement. Therefore, it is important to recognize this disease and be aware of underdiagnosis because of emphasis on pathologic evidence. Here, we present a case of allergic desensitization causing Churg-Strauss syndrome in the absence of other known factors.

## Introduction

Churg-Strauss syndrome (CSS) is a vasculitis of medium to small sized muscular arteries and veins. CSS was first described in 13 patients with deadly asthma who had the combination symptoms of asthma and eosinophilia, rhinosinusitis and signs of vasculitis in the heart, pericardium, the nervous and gastroenteric systems and other vital organs [1]. Even though some consider allergic asthma, rhinosinusitis and eosinophilia separate of the CSS, most reports consider this vasculitis a disease by itself or a variant of asthma which occurs from immune system involvement such as with the use of medications like leukotrienes or sudden withdrawal of oral steroids [2,3]. They also consider environmental factors significant in development of primary systemic vasculitides [4]. One of these factors is the influence of severe antigenemia (SAg) in inducing vasculitis [3,5]. Presumptively inhaling allergens, vaccination, desensitizing medications and even infections and Loeffler's syndrome can behave as a triggering event against T-lymphocytes and cause the signs of CSS in the asthmatic population [4,6,7].

## Case presentation

The patient was a 19-year-old Persian male with a past medical history of sinusitis and allergic rhinitis for four years and episodes of wheezing and shortness of breath for two years who was admitted for fever, productive cough, worsening of dyspnea in the past two months. Two weeks prior to admission, in addition to increased shortness of breath, blood streaks were present in his sputum and he developed gradual paresthesia in his right lower legs. On physical examination, he was in respiratory distress and had coarse crackles and generalized ronchi and wheezing in the lung bases. The level of SPO_2 _upon admission was 74% and increased to 91% with administration of 6 liters of 40% oxygen via venturi mask. Our patient's hemodynamics were stable except for tachypnea and he was not febrile. A complete blood count showed a white cell count (WBC) of 18,000 with 23% eosinophils with no anemia and the platelet count was 215,000 per cubic millimeter. Liver and renal function tests, blood coagulation studies and electrolytes were within normal limits. Our patient's erythrocyte sedimentation rate (ESR) was 3mm/h 15 days prior to admission and 14mm/h at the hospital. Blood cultures were negative one week after admission. Urinalysis was normal. Tests for rheumatoid factor (RF), anti nuclear antibodies (ANA), circulating anti neutrophil cytoplasmic antibody (cANCA), perinuclear anti neutrophil cytoplasmic antibody (pANCA) as well as C3, C4 and CH50 were negative. Skin tuberculin test (PPD) was negative at 72 hours. Cardiac echography was normal. On the second hospital day, our patient developed hyperesthesia and severe pain in his right lower leg which led to a foot drop on the same side the following day. He underwent EMG (electromyography) and NCV (nerve conduction study) studies that showed neuritis and axonopathy along the common peroneal nerve located below the right knee level. He eventually underwent bronchoscopy and transbronchial lung biopsy (TBLB) of the right lower lobe and of mucosa of trachea and bronchi. Lung pathology showed infiltration with eosinophilic and limited number of multinucleated giant cells. Bronchi and lung parenchyma were free of signs of vasculitis but accumulation of eosinophils in the alveoli was seen. Bronchoalveolar lavage (BAL) revealed neither mycobacteria nor cancerous cells. A closer look at our patient's past medical history revealed desensitization vaccination for allergy control. The vaccines were first administered twice weekly, every Monday and every Wednesday for four months and again this year, for a period of three months. He had no history of switching inhaled steroid medications to oral steroids or the use of leukotriene antagonists.

During his hospital stay, our patient was treated with oral steroids and cyclophosphamide and he was discharged two weeks after admission in no respiratory distress and good general health.

## Discussion

CSS is unknown in origin. Suggestions are allergic or immune mediated. Etiology is considered to be autoimmune due to allergic symptoms, immune complex mediation 48% are (ANCA, antineutrophil cytoplasmic antibody) positive, increased T-cell mediated immunity, elevated immunoglobulin E (IgE) levels and rheumatoid factor [6]. Also superantigen theory proposes that due to SAg, vascular wall destruction by autoreative T-cells may occur or autoreactive B-cells may form antibodies involved in formation of vasculitis [5]. The latter has been considered associated with cases of sudden loss of control over immune reaction as with stopping steroids, leukotriene antagonists or in this case probably due to invigoration of the immune system with antigen stimulation. Here, we have presented the case of allergic desensitization causing CSS in the absence of other known factors.

Clinical diagnosis is based on American College of Rheumatology Criteria which requires the presence of four or more of the six conditions: asthma, eosinophilia > 10%, neuropathy, migratory or transient pulmonary opacities, abnormalities of the paranasal sinuses and extra-vascular eosinophils on biopsy. Regularly, a history of asthma and rhinosinusitis precedes the appearance of the syndrome itself [8].

Histological diagnosis is by demonstration of vasculitis that is necrotizing, tissue infiltration with eosinophils and extra-vascular granulomas are found in a few cases [9].

Asthma is pivotal to the diagnosis and is relatively late in age of onset. The most common radiological findings are lobular or centrilobular peripheral consolidation and nodularity, thickened bronchial walls, inter-lobular septum, and peri-cardial and pleural effusion [8]. On computed tomography, enlargement of or distortion of peripheral pulmonary arteries may be seen. The ultimate diagnosis is by biopsy with open biopsy preferred to TBLB or sural nerve sampling in case of symptoms [10].

The differential diagnosis includes Wegener's granulomatosis, drug reaction, bronchogenic granulomatosis, fungal and parasitic infections, and malignancy [6]. Included in the differential diagnosis are immune mediated diseases such as idiopathic thrombocytopenic purpura, cryoglobulenemia, and collagen vascular diseases. Complement is normal or elevated in vasculitis and malignancy generally does not cause failure of major organs [10]. Polyarteritis nodosa (PAN) also involves small and medium sized vessels, and severe renal disease is common, but eosinophilia is rare [2]. Diagnosis is by clinical, pathological, and laboratory correlates.

In the case of our patient, asthma was present years before the disease. The clinical criteria for diagnosis of CSS were met as well as eosinophilia. Other organ involvement was cardiac and neurological. Respiratory symptoms responded well to oral corticosteroids and cyclophosphamide. Allergic angiitis had not occurred after the first episode of desensitization treatment, although he might have had a mild reaction for which medical treatment was not saught.

Treatment regularly is with prednisone starting at 40-60 mg a day and the occasional addition of cyclophosphamide or azathioprine with the purpose to limit the disease or spare steroids [2]. In case of fulminant disease or multi-organ involvement, parenteral corticosteroid such as methylprednisolone is used. If response to mentioned treatment is not seen, parenteral immunoglobulin is administered. Newly experimental medications for unresponsive cases are mycophenolate mofetil and tumor necrosis factor (TNF)-α blockers (such as etanercept and infliximab). Treatment is continued at least one month after remission [1,11]. Uncommon but presented as case report has been also cardiac function improvement (ejection fraction increased from 28 to 67%) after a year of corticosteroid therapy [12].

Hyper-responsiveness to antigenic stimuli may be the etiologic factor in the development of CSS. In 1998, Wechsler and colleagues reported eight patients with this syndrome after a decrease or withdrawal of oral steroids. Subsequent to this report, many others were made that associated CSS with the withdrawal of leukotriene antagonists or systemic steroids and this strengthened the theory of unmasking of CSS in chronic and allergic asthma [2]. Also cases have been attributed to antibiotics such as erythromycin, azithromycin and roxithomycin and some metered dose inhalers (bamuterol, salmeterol and nedrocromil) as well as the Food and Drug Administration mentioned fluticasone [1]. Vasculitis has also been reported with hepatitis B, flu and measles vaccine and BCG (Bacillus of Calmette and Guerin) inoculation. Also to be considered is vaccine components such as thimerosol or aluminum which have not been shown to have many side effects [13,14]. Finally, CSS has been associated with hyposensitizing immunization to candidin, polymicrobial and acarian or unspecific antigens [7]. In cases of PAN which has extreme closeness to CSS, similar reports with hyposensitization have been observed. In one study, 20 patients with PAN developed vasculitic symptoms upon hyposensitization treatment for atopic (IgE-mediated) lung disease. Allergic symptoms were present in patients from less than three months to 10 years. Despite withdrawal of treatment, vasculitis persisted. Death resulted in three patients. Fourteen control PAN patients (without hyposensitization) had less skin involvement and peripheral eosinophilia (P < 0.05). Signs of circulating immune complexes and decreased hemolytic complements, cryoglobulinemia and elevated C1q binding were present in all subjects. Allergen use was not of one type; five patients received less than 16 mg of allergenic protein and anti-allergen precipitating antibodies were not found [15].

CSS might be under diagnosed because of a focus on pathologic evidence. Classic histology is seldom found and is not pathognomonic for diagnosis. Yet, the disease has distinctive clinical presentation and criteria developed show the importance of this. It is important to diagnose the disease and monitor for relapse among these patients as it is frequent and requires immediate attention. Additionally, despite treatment of vasculitis, neurological symptoms may persist [6].

## Conclusions

Although the etiology of this disease is not well known, both T- and B-cell mediated immunity have been considered. As noted in this case and many others, etiologies for vasculitic syndromes and CSS are various and medications are among them. Cases related to medications are rare as is the disease incidence itself. A unifying etiology for disease occurrence can not be distinguished. Maybe medication related CSS might be related to a drug preparation technique such as the additives thimerosol or aluminum. Yet, this disease is considered immune mediated and autoimmune mediated and has been seen in other situations particularly infections, due to environmental factors it might have multiple etiologies [6].

According to our findings, CSS seems to be developed after cessation of allergic desensitization vaccination; however more investigations should be carried out.

## Consent

Written informed consent was obtained from the patient for publication of this case report and any accompanying images. A copy of the written consent is available for review from the Editor in Chief of the journal.

## Competing interests

The authors declare that they have no competing interests.

## Authors' contributions

MRM, SFT, AC, and NF participated in planning of case reporting and provided case presentation and guidance and manuscript was prepared by all authors including FT and BM. All authors read and approved the final manuscript.

## Acknowledgements

Authors would like to thank all colleagues at the Masih Daneshvari Hospital that helped with preparation of this manuscript.
